# Hyaluronic Acid in Nanopharmaceuticals: An Overview

**DOI:** 10.3390/cimb46090621

**Published:** 2024-09-20

**Authors:** Sina Matalqah, Zainab Lafi, Sara Yousef Asha

**Affiliations:** 1 Pharmacological and Diagnostic Research Center, Faculty of Pharmacy, Al-Ahliyya Amman University, Amman 19328, Jordan; 2 School of Medicine, University of Jordan, Amman 19328, Jordan

**Keywords:** nanotechnology, hyaluronic acid, nanopharmaceuticals, lubricant

## Abstract

Hyaluronic acid (HA) is a naturally occurring, long, unbranched polysaccharide that plays a critical role in maintaining skin structure and hydration. Its unique properties make it a valuable component in the field of nanopharmaceuticals. The combination of HA into nanopharmaceuticals enhances its ability to interact with various therapeutic agents, improving the delivery and efficacy of drugs. HA-based nanoparticles, including solid lipid nanoparticles, and polymeric nanogels, offer controlled release, enhanced stability, and targeted delivery of therapeutic agents. These innovations significantly improve therapeutic outcomes and reduce side effects, making HA an essential tool in modern medicine. In general, HA-modified liposomes enhance drug encapsulation and targeting, while HA-modified solid lipid nanoparticles (SLNs) provide a solid lipid core for drug encapsulation, offering controlled release and stability. This article provides an overview of the potential applications and recent advancements of HA in nanopharmaceuticals, emphasizing its significant impact on the evolving field of targeted drug delivery and advanced therapeutic strategies. By delving into the unique properties of HA and its compatibility with various therapeutic agents, this review underscores the promising potential of HA in revolutionizing nanopharmaceuticals.

## 1. Introduction

Hyaluronic acid (HA) is a remarkable biopolymer that has surpassed its popularity in cosmetic applications to become a cornerstone in advanced medical treatments. Known for its excellent biocompatibility, biodegradability, and non-immunogenic properties, HA is increasingly utilized in the development of sophisticated drug delivery systems. This review explores the latest advancements in HA-based technologies, with a particular focus on their application in ocular drug delivery and other medical fields [1]. Hyaluronic acid (HA) is a powerhouse in the world of biomedical applications, especially in drug delivery systems. HA was recognized for its exceptional biocompatibility, biodegradability, and non-immunogenicity, and has been making waves in medical research and treatments. From enhancing drug targeting in cancer therapies to improving the delivery and efficacy of ocular medications, HA technologies are at the forefront of innovative healthcare solutions [2].

**Figure 1:**
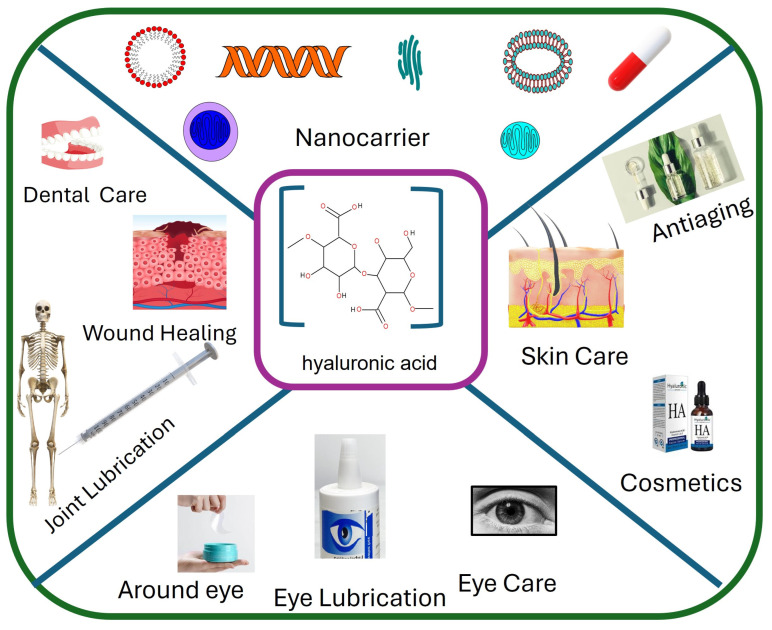
Scheme for different applications of hyaluronic acid.

HA is a naturally occurring polysaccharide found in various applications and tissues of the body, including the skin, eyes, surgery, cosmetics, and joints, as in Table 1. Its ability to retain water and form viscoelastic gels makes it ideal for medical applications. In recent years, researchers have harnessed these properties to develop HA-based nanoparticles that enhance drug delivery efficiency and effectiveness. These nanoparticles can encapsulate therapeutic agents, protecting them from degradation and controlling their release over time [3]. Hyaluronic acid’s versatility and natural compatibility with the human body make it a valuable tool across multiple medical fields (Figure 1). Its ability to enhance healing, provide lubrication, and maintain hydration underscores its importance in both therapeutic and cosmetic applications, improving patient outcomes and quality of life [4].

Hyaluronic acid is a key component in the treatment of osteoarthritis, especially in the knees. HA injections, often referred to as viscosupplementation, help lubricate the joint, reduce pain, and improve mobility. This treatment can provide relief for several months, offering a non-surgical option for managing arthritis symptoms [5].

Additionally, HA is extensively used in wound care due to its ability to promote tissue regeneration and accelerate healing. It helps maintain a moist environment, which is essential for wound healing, and has anti-inflammatory properties that reduce pain and swelling. HA-based dressings and gels are commonly applied to burns, ulcers, and surgical wounds [6]. In eye surgery, such as cataract removal and corneal transplantation, HA is used as a viscoelastic agent. It helps to maintain eye shape, protect delicate tissues, and facilitate the surgical process. Additionally, HA is a key ingredient in many eye drops, providing long-lasting moisture and relief for dry eyes [7].

Hyaluronic acid is a star ingredient in the cosmetic industry, particularly in dermal fillers. These fillers are injected into the skin to reduce wrinkles, add volume, and enhance facial contours. HA’s hydrating properties make it an excellent choice for skin care products, as it can hold up to 1000 times its weight in water, keeping the skin plump and youthful [4].

HA is used in treatments for gastrointestinal conditions, such as acid reflux and gastritis. It helps protect and repair the lining of the stomach and intestines, reducing inflammation and promoting healing [8].

In dental care, HA is utilized to promote gum health and healing after surgical procedures. It helps reduce inflammation, supports tissue regeneration, and can be found in various oral care products, such as mouthwashes and gels [9].

**Table 1:** Summarizing the medical applications of HA.

Medical Field	Application	Description	References
Dermatology	Skin moisturization	HA is widely used in topical formulations (creams, serums) to hydrate and improve skin elasticity.	[10,11]
	Dermal fillers	Injected as a filler to reduce wrinkles and enhance facial volume in cosmetic treatments.	[12]
	Wound healing	Used in wound dressings to promote tissue repair and hydration of chronic wounds.	[13,14,15,16,17,18]
Ophthalmology	Ocular lubricants	Found in artificial tears and eye drops to treat dry eyes and enhance comfort during ocular surgeries.	[19,20,21]
	Eye surgery	Used during cataract surgery and other ocular procedures to maintain eye structure and protect tissues.	[22,23,24,25,26,27]
Orthopedics	Osteoarthritis treatment	Injected into joints (e.g., knee) as a viscosupplementation to lubricate and reduce pain associated with osteoarthritis.	[28]
	Cartilage regeneration	Investigated as a scaffold material for cartilage repair and tissue engineering.	[29,30,31]
Aesthetic Medicine	Facial rejuvenation	Injected for skin hydration, volume restoration, and anti-aging effects in aesthetic treatments.	[32,33,34]
Rheumatology	Pain management in joint disorders	Intra-articular injections used to relieve pain and improve mobility in rheumatoid arthritis and joint disorders.	[28,35,36]
Surgery	Post-surgical adhesion Prevention	Applied in gels to reduce the formation of adhesions after surgeries, particularly in abdominal and pelvic operations.	[37,38,39]
Dentistry	Periodontal therapy	Applied in periodontal treatments to promote healing of gum tissues and reduce inflammation.	[40,41,42]

Recent studies have demonstrated the potential of HA-based drug delivery systems in improving the bioavailability and therapeutic outcomes of ocular medications. By forming a protective barrier, HA nanoparticles can prolong the retention time of drugs in the ocular environment, reducing the need for frequent administration and minimizing side effects.

In cancer therapy, HA nanoparticles are being developed to target tumor cells specifically, minimizing damage to healthy tissues. The unique affinity of HA for certain cell surface receptors, such as CD44, which is overexpressed in many cancer cells, allows for more precise drug targeting. This precision reduces systemic toxicity and enhances the overall effectiveness of the treatment.

HA’s applications are also expanding into the field of respiratory medicine. Researchers are exploring the use of HA-based systems to deliver drugs to the lungs, offering potential treatments for certain diseases, like asthma and chronic obstructive pulmonary disease (COPD). The ability of HA to form hydrogels that can be inhaled opens new avenues for non-invasive drug delivery, improving patient outcomes and adherence to treatment protocols.

The development of HA-coated nanoparticles represents a significant advancement in drug delivery technology. These nanoparticles combine the unique properties of HA with the stability and biocompatibility of nanoparticles, creating a highly effective delivery system for poorly water-soluble drugs. Such systems can protect drugs from harsh gastric conditions, ensuring their release in more favorable intestinal environments [43].

The synthesis method for creating these nanoparticles simplifies the production process, making it more feasible for large-scale manufacturing. This approach reduces the number of steps involved, minimizes material loss, and improves the overall efficiency of nanoparticle production. The result is a more practical and scalable solution for developing advanced drug delivery systems [1].

HA has been extensively studied for its role in skincare, tissue repair, and drug delivery, with many reviews covering its biological properties and uses. However, this review takes a deeper dive into how HA is being incorporated into advanced nanopharmaceuticals. Unlike earlier reviews that focused on HA’s general functions, this article explores the exciting innovations in HA-based nanoparticles. These new formulations enhance drug delivery, improve stability, and allow for controlled release, offering significant benefits for patients. By highlighting the practical and cutting-edge applications of HA in drug delivery systems, this review provides a fresh look at HA’s increasing importance in modern medicine, especially in targeted therapies.

## 2. Structural Features and Properties of Hyaluronic Acid

**Figure 2:**
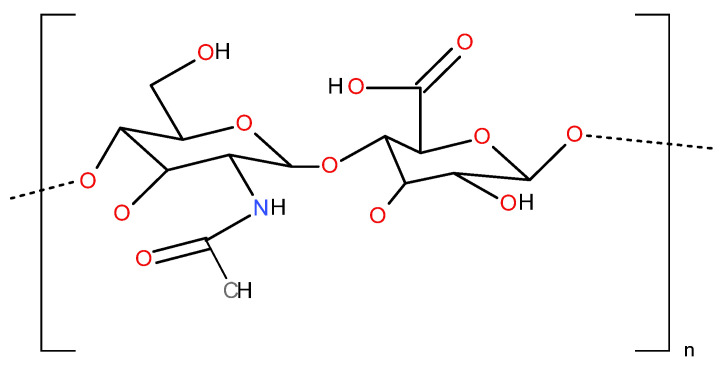
Hyaluronic acid’s structure.

Hyaluronic acid (HA) is a naturally occurring linear, unbranching disaccharide composed of repeating units of glucuronic acid and N-acetyl glycosamine joined alternatively by β 1–3 and β 1–4 glycoside bonds with molecular weight (MW) values reaching up to 2 × 10^7^ Da. Each repeating disaccharide unit has one carboxylate group, four hydroxyl groups, and an acetamido group, as shown in (Figure 2). HA has a negative charge and is hydrophilic due to the presence of carboxyl groups. HA was isolated from the vitreous body of the bovine eye by Karl Meyer and John Palmer in 1934, but its structure was described by Laurent in 1970. In 1986 Balazs et al. reported that “hyaluronan” is an alternative to “hyaluronic acid”, since it does not have sulfate group and the carboxyl groups are dissociated and so attract cations, e.g., Na+ and form sodium hyaluronate at physiological pH. Hyaluronan has a unique characteristic: its solution manifests very unusual rheological properties, and it is exceedingly lubricious and very hydrophilic [44].

### 2.1. Hyaluronic Acid’s Molecular Weight and Its Uses

Hyaluronic acid (HA) is a versatile molecule available in various molecular weights, each suited for specific applications. Low molecular weight HA (5–20 kDa) penetrates the skin more easily, making it highly effective in skincare products for anti-aging, as it helps reduce fine lines and wrinkles by promoting hydration and collagen synthesis [45,46]. It is also beneficial for wound healing, accelerating the process by promoting cell proliferation and migration, and is used in drug delivery systems due to its efficient delivery through the skin or mucous membranes. Medium molecular weight HA (20–200 kDa) balances penetration and moisture retention, making it ideal for skincare products to provide hydration and improve skin texture and elasticity [43]. It is also used in ophthalmology for treating dry eye syndrome and lubricating the eyes during surgeries, as well as in joint health treatments for osteoarthritis, acting as a lubricant and shock absorber to reduce pain and improve mobility [47,48]. High molecular weight HA (over 200 kDa) forms a protective barrier on the skin or tissues, making it perfect for cosmetic fillers to provide volume and fullness, reduce deep wrinkles, and enhance facial contours. It is also used in lubrication supplements for joint disorders, restoring the viscoelastic properties of synovial fluid, and in skincare for long-lasting hydration by forming a film on the skin’s surface to prevent moisture loss. Understanding these different molecular weights and their specific uses allows for targeted and effective applications in medical and cosmetic fields [49,50]

### 2.2. Synthesis and Degradation of Hyaluronic Acid

**Figure 3:**
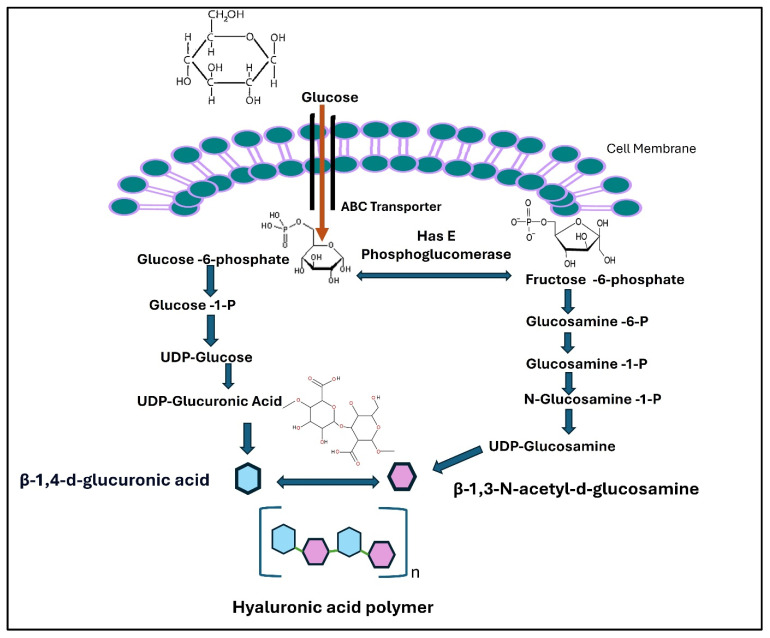
Schematic representation of hyaluronic acid synthesis.

The three transmembrane isoenzymes responsible for HA synthesis are HA synthetase 1 (HAS1), HA synthetase 2 (HAS2), and HA synthetase 3 (HAS3) [51]. They catalyzed the formation of HA by polymerizing UDP-N acetylglucosamine and UDP-glucuronic acid (Figure 3) in the presence of Mg^2+^ or Mn^2+^. HA is synthesized at the inner surface of the plasma membrane, and then the molecules are moved through a pore within the HAS structures to the extracellular space with the elongation of the polymeric chain [52].

### 2.3. The Mechanism of Action of HA and Its Biological Function

HA is degraded by the two mechanisms, i.e., enzymatically (hyaluronidase, β-D-glucuronidase, and β-N-acetyl-hexosaminidase) or by free radicals [53]. These hydrolytic enzymes are found throughout the body in various forms, intracellularly and in serum, and they cleave HA into oligosaccharides while β-d-glucuronidase and β-N-acetyl hexosaminidase enable further degradation by removing nonreducing terminal sugars [54]. HA turnover in tissues can occur through local degradation or by being released into the lymphatic systems. The half-life of HA in human tissue can vary, ranging from about 1 day in the skin layer to up to 70 days in the vitreous body of the eye [55].

Depending on the molecule size, HA works via two different mechanisms, namely as a passive structural molecule or as signaling molecule. The passive structural effect of the HA depends on its physicochemical properties, such as hygroscopicity and viscoelasticity, and involve improving hydration, water balance, and structural integrity [56].

While the signaling action of HA depends on its molecular weight (MW), location, and particular cell factors (receptor expression, signaling pathways, and cell cycle), the interaction between HA and its proteins determines opposing actions. These actions include pro- and anti-inflammatory activities, the promotion and inhibition of migration activation, and the blocking of cell division and differentiation [57]. Moreover, HA can modify immune cell function through interactions with cytokines [58]. HA produces its therapeutic effects by binding to three main cell surface receptor types: CD44 (a membrane glycoprotein), RHAMM (the receptor for hyaluronate-mediated motility), and ICAM−1 (intercellular adhesion molecule 1) [59]. As these molecules are non-homologous proteins, they have the potential to cause reactions in cells other than those specific to the cell surface on which they are located.

CD44 has been extensively studied as an HA receptor, and the effectiveness of HA as a signaling molecule is highly dependent on the affinity of CD44 for HA. RHAMM, also known as CD168, is another surface receptor for HA found in several cell types. Through interactions with skeletal proteins, it facilitates cell migration, particularly during processes related to inflammation and tissue repair [60]. Also, this receptor has been found to be intracellularly linked to mitochondria, microtubules, and nuclei, as well as extracellularly, where it associates with CD−44 receptor [61].

### 2.4. Derivatives of HA

HA can be chemically modified by conjugation or crosslinking reactions using the available functional groups (carboxylic acid and hydroxyl groups) to produce HA derivatives that possess an improved natural turnover and clearance compared with the HA. Also, the HA–drug bioconjugates possess an improved water solubility and affinity to receptors compared to the parent drug.

A series of HA derivatives with an improved resistance to degradation compared to native HA have been produced, among these the derivative bearing ethylenediamine (EDA) and octadecyl amine (C 18 -NH 2) named HA-EDA-C 18. Fabio S. Palumbo et al. exploited the hydrophobic properties of HA-EDA-C 18 to control the release of dexamethasone as chondrogenic stimulator [62].

Mauro Pavan et al. synthesized an intra-articular alkyl derivative of HA. This derivative displayed an unpredictable biological activity against two enzymes that are involved in joint inflammation: ChC for MMPs (a gene that encode several inflammatory proteins and induce the activation of cartilage-degrading enzymes) and BTH for hyaluronidases. The results showed that such derivatives can act as MMP and hyaluronidase inhibitors for use in osteoarthritis (OA) treatment [63]

Sannino et al. synthesized chemically crosslinked hydrogels from HA, water solutions of hydroxyethyl cellulose (HEC), and carboxymethylcellulose sodium salt (CMCNa) and (HA), using a non-toxic water-soluble carbodiimide (WSC) as a crosslinking agent. They found that these hydrogels are potentially biodegradable and biocompatible, eco-friendly, and show high absorbent activity. This material was tested as a bulking agent, and the preliminary results are encouraging [64].

Other adipic acid dihydrazide (ADH)-modified hyaluronic acid (HA-ADH) derivatives were synthesized by Chien-Ming Hsieh et al. and conjugated with QDots (QDots-HA conjugates) to study the effects of the molecular weight (MW) and extent of chemical modification of HA on its biodistribution. Results showed that QDot-HA conjugates with a higher MW of HA or high modification showed slow clearance leading to an extension of the retention time for up to 10 days, whereas those with lower MWs of HA or low modification exhibited quick absorption and elimination after oral administration [65].

## 3. Nanopharmaceuticals: Enhancing Drug Delivery

Nanopharmaceuticals are a new class of therapeutic agents containing nanomaterials that often have unique nano-physiochemical properties due to their small size, large surface area, and the possibility of adjusting their properties.

Originally, they were nanoparticles (NPs) designed to be used in a wide range of clinical therapeutic applications. Nanopharmaceuticals can be used in organ or tissue targeting, which in turn reduces toxicity and enhances bioavailability of the therapeutic agent and so increases its efficacy and patient compliance. Also, these nanopharmaceuticals can also increase the half-life of the therapeutic agent by decreasing its metabolism. The advantages of these nanopharmaceuticals are outlined in Table 2. With these advantages, nanopharmaceuticals could extend the economic life of patented drugs, generating new sources of income.

A significant number of nanopharmaceuticals that have been approved by the US Food and Drug Administration (FDA) are already on the market, and many others are just waiting to be approved by regulators.

## 4. Types of Hyaluronic Acid-Based Nanoparticles

### 4.1. Hyaluronic Acid-Modified Liposomes

Hyaluronic acid-modified liposomes are used to improve the efficiency of drug encapsulation and to enhance targeting ability. By coating liposomes with hyaluronic acid, they can better adhere to and penetrate specific cells, making them highly effective for targeted drug delivery [88].

### 4.2. Hyaluronic Acid-Modified Solid Lipid Nanoparticles (SLNs)

These nanoparticles have a solid lipid core that encapsulates drugs, providing controlled release and enhanced stability. The solid lipid matrix protects the encapsulated drug from degradation and allows for a sustained release profile, which can be tailored to specific therapeutic needs. The modification of SLNs with hyaluronic acid further enhances their targeting ability, enabling them to bind specifically to receptors on target cells, such as cancer cells or inflamed tissues [89]

### 4.3. Hyaluronic Acid-Modified Nanostructured Lipid Carriers (NLCs)

Hyaluronic acid-modified nanostructured lipid carriers (NLCs) consist of a blend of solid and liquid lipids, which allows for higher drug-loading capacity and improved release profiles. This hybrid structure leverages the benefits of both solid and liquid lipid phases to enhance drug solubility and stability [90].

### 4.4. Hyaluronic Acid-Based Nanogels

Polymer micelles linked with hyaluronic acid are designed to solubilize hydrophobic drugs, enhancing their delivery to specific sites in the body. The micelle structure, with its hydrophobic core and hydrophilic shell, allows these carriers to encapsulate poorly soluble drugs and protect them from degradation. The addition of hyaluronic acid not only improves the stability and circulation time of these micelles but also enhances their ability to target specific cells, such as cancer cells, through receptor-mediated endocytosis. This targeted approach ensures that a higher concentration of the drug reaches the diseased site, reducing side effects and improving therapeutic outcomes. These micelles are particularly valuable in cancer treatment, where the precise delivery of chemotherapy agents is crucial for effectiveness and minimizing harm to healthy tissues.

### 4.5. Hyaluronic Acid-Coated Nanoparticles

Hyaluronic acid-coated nanoparticles represent an exciting and innovative advancement in the field of nanopharmaceuticals. By coating nanoparticles with hyaluronic acid (HA), researchers have developed a versatile delivery system that offers numerous benefits for medical applications.

When introduced into the body, these nanoparticles are less likely to be recognized and attacked by the immune system. This reduced immune response helps in maintaining the stability and longevity of the nanoparticles in the bloodstream, allowing them to reach their intended targets more effectively [91,92].

**Figure 4:**
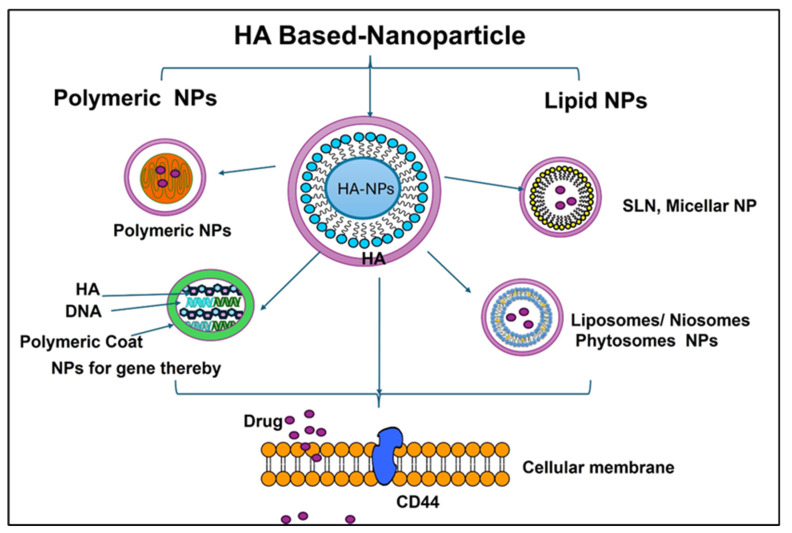
Schematic illustration of hyaluronic acid-based nanoparticles, showing their targeted ability through a CD44 receptor.

One of the most significant advantages of HA-coated nanoparticles is their enhanced targeting capability [93]. HA has a natural affinity for certain cell receptors, particularly those overexpressed in cancer cells and inflamed tissues (Figure 4). This means that HA-coated nanoparticles can selectively bind to these receptors, ensuring that the therapeutic agents they carry are delivered directly to the diseased cells [94].

In cancer therapy, for instance, they can deliver chemotherapy drugs directly to tumor cells, sparing healthy tissues and reducing adverse effects. Additionally, in anti-inflammatory treatments, HA-coated nanoparticles can target inflamed tissues, delivering anti-inflammatory drugs precisely where they are needed. The versatility of HA-coated nanoparticles extends to their ability to carry a wide range of therapeutic agents, including small-molecule drugs, proteins, and nucleic acids. This adaptability makes them suitable for treating various diseases and conditions. Moreover, the HA coating can be engineered to respond to specific stimuli, such as changes in pH or temperature, allowing for the controlled and sustained release of therapeutic agents [95].

### 4.6. Role of Hyaluronic Acid in Enhancing Delivery Efficiency

Due to its unique properties, HA has received much attention as a tool for developing drug delivery systems. HA was used as a carrier in drug delivery in parenteral and non- parenteral routes. Parentally, HA was useful for sustained release formulations of protein and peptide drugs, since conventional drug delivery systems using polylactic glycolic acid (PLGA) produce inflammation and protein denaturation associated with the degradation of PLGA [96].

OFL Johnson et al. [97] prepared an injectable sustained–release form of human growth hormone without altering its integrity using polylactic glycolic acid (PLGA) in 1999 in the United States. However, this formula was withdrawn from the market due to the side effects associated with it included scale-up difficulties, pain from the injection, and the denaturation of human growth hormone because of the hydrophobic interaction with PLGA and the acidic environment that resulted from PLGA disintegrating. As a result, HA has received increased attention for developing a sustained-release formula of human growth hormone [97].

HA also can promote tumor targeting, via either active targeting through binding to CD44 and other cell surface receptors or passive targeting through enhanced permeation retention phenomena (EPR) [98].

Wang T et al. prepared HA-coated chitosan NPs to encapsulate 5-fluorouracil to enhance drug accumulation in tumor cells and to improve its antitumor efficiency by offering targeted drug delivery via CD44 [99]. Due to the physiochemical properties of HA (water solubility, elasticity, and biocompatibility) it is widely used non-parentally, including in ocular and nasal delivery. Bernatchez et al. reported that the bioavailability of gentamicin can be increased when formulated with a 0.25% HA solution due to an increase in the ocular residence time because of its mucoadhesive property [100]. Additionally, Hume et al. prepared a sustained ocular prednisolone formula by using HA benzyl ester films [101].

Also, HA can be used intranasally due to its mucoadhesive properties, which enhance the absorption of drugs and proteins via mucosal tissues. A formula of HA-based microspheres containing PEG 6000 and/or sodium taurocholate was prepared by the spray dry method for the nasal delivery of fexofenadine hydrochloride, which enhances the AUC and Cmax values [102]. Moreover, Singh et al. prepared an effective a bioadhesive system comprised of esterified hyaluronic acid microspheres in combination with a mucosal adjuvant (LTK63) for the intranasal administration of a flu vaccine [103].

## 5. Compatibility with Therapeutic Agents

Hyaluronic acid (HA) is widely used in topical applications as a moisturizing and wound healing agent. Hyaluronic acid promotes wound healing through the inhibition of oxidative stress in wounds, so formulations that contain HA with a carrier to locally deliver active biomolecules, such as antioxidants, are of great interest to assist the healing of wounds.

Pooyan Makvandi et al. [104] developed an HA-based formula with antioxidant activity containing vitamin E and natural compounds, such as raspberry extract and green tea for potential topical applications, targeting wound healing. They reported that this formula showed better wound healing and antioxidant activity than the control formulation and that the presence of HA had dual actions, namely enhancing the viscoelastic properties to allow the topical use of this formula and improving the wound healing properties with the antioxidant action of vitamin E and natural compounds [104].

Furthermore, Richard D. et al. investigated the role of HA in the wound healing process. According to their report, HA has important rheostatic- and viscosity-controlling properties as a macromolecule, and its degradation products have important modulating effects on the healing wound. It increases the angiogenic response from the wound bed as well as keratinocyte migration and proliferation [105].

Dry eye disease (DED) is a common pathological disease with a high prevalence that usually requires continued treatment. Using lacrimal substitutes composed of polymers and osmoprotectant agents is the first line in DED treatment. Also, the combination of HA, carmellose, and osmoprotectors are effective in the treatment of symptoms and signs of DED due to the synergistic effects of all its components, as reported by Mateo et al. [106].

Cristache et al. investigated the anti-inflammatory activity of combining melatonin (MEL) and HA in a complex active compound on periodontal structures and repair mechanism and the possible synergic effect and if each component of the proposed mixture (MEL-HA) would keep its specific characteristics, when using both active principles, for a topical application. They found that there are many positive effects of MEL, as well as for HA, and the use of individual compounds on the periodontium and in the formula were very efficient for the treatment of dental wounds [107].

On the other hand, different topical applications of 0.1% hyaluronan formulations with different molecular weights (MW) were tested as anti-wrinkle agent by Pavicic T et al., who found that all of the HA-based creams prepared showed a significant improvement in skin hydration when compared to a placebo after 60 days of treatment [108].

Osteoarthritis (OA) is a common inflammatory disease and the main cause of disability worldwide, and it affects the population over the age of 50. Physiotherapy, pain management, and joint replacement were the only available effective treatments for OA. Chen et al. reported that the combination of HA and platelet-rich plasma (PRP) treatment could be recognized as an intra-articularly injectable, regenerative, and anti-inflammatory agents for future clinical OA therapy [109].

## 6. Recent Advancements and Innovations

Due to its unique properties, HA has been widely used in biomedical application and as a carrier for receptor-mediated drug targeting in cancer therapy and for the delivery of protein, peptide, and imaging agents due to its ability to recognize receptors whose expression is increased in various diseased cells.

In the last few years, many advances concerning the application of HA and its derivatives in drug delivery have been made, starting from the formation of hydrogels by crosslinking reactions of HA to combining HA with various polymers (coating or conjugation) or combining it with polycations to form polyelectrolytes for cancer and gene therapy, as well as for the diagnosis of different diseases.

Mangano, K et al. conjugated P40 (a fragment isolated from Corynebacterium granulosum, which possess antitumor, antibacterial, phagocytic, and antiviral functions, as well as cytokine induction effects) with hyaluronic acid to assess dermatitis induced by oxazolone in a mouse model. Hyaluronic acid–P40 conjugate cream or placebo-containing cream were used topically to treat the inner and outer surface of the left ear of mice after the sensitization. The results showed a significant reduction in ear thickness and weight and in edema and leukocyte recruitment in the mice treated with hyaluronic–P40 conjugate cream compared with mice treated with the cream base alone [110].

As mentioned above, HA is a negatively charged polysaccharide, so it can interact with positively charged species to form polyion complexes to be used as carriers for drug delivery and target therapy in the form of hydrogels and microparticles. Skorik et al. studied the interaction between diethylamino ethyl chitosan and HA to form spherical colloidal nanoparticles and used it as a carrier for colistin delivery. Colistin is a positively charged antibiotic which can interact with anionic molecules, forming negatively charged nanoparticles, and which shows a good activity on multidrug-resistant Gram-negative bacteria. HA has been presented in several pharmaceutical forms, including nanoparticles (NPs), nanocomplexes, matrices, and hydrogels.

It is worth mention that a major limitation of HA in drug delivery is its rapid biodegradability, which reduces its therapeutic effectiveness, and the challenge of preserving its natural biocompatibility when attempting chemical modifications to improve stability must be considered [111,112].

## 7. Challenges and Limitation

A big issue with HA is its stability. HA is naturally broken down by the enzyme hyaluronidase, which limits how long it can effectively carry and release drugs. Chemical modifications, like crosslinking can improve stability, but these changes might reduce its natural compatibility with the body, leading to unwanted side effects. Finding a way to make HA more stable without affecting its safety is a key challenge [112,113,114].

Another problem is its drug loading efficiency, especially with hydrophobic (water-repelling) drugs, including many of those used in cancer treatment. HA’s hydrophilic (water-attracting) nature makes it unable to hold and deliver these drugs effectively. While scientists have tried solutions, like attaching hydrophobic groups to HA, these can complicate drug release and lower the amount of drug that reaches the target [115,116].

Tissue penetration is another challenge. While HA binds well to CD44 receptors in cancer cells, its large molecular size makes it unable to penetrate deeply into tissues, like solid tumors. Reducing HA’s size helps it reach deeper tissues, but this also weakens its ability to target specific cells, creating a difficult trade-off between depth and precision.

Immune responses are also a concern, especially when HA is chemically modified. While natural HA is well-tolerated by the body, modified versions can trigger immune reactions, like inflammation or allergic responses, particularly with repeated use. Maintaining functionality while minimizing immune reactions is crucial for long-term safety [117].

On the industrial side, scaling up the production of HA-based systems is difficult due to the natural variability in HA based on its source and extraction process. This affects the quality and performance of the final product. Also, the chemical processes needed to modify HA are often costly and complex, making large-scale production expensive and labor-intensive [118].

Finally, regulatory challenges add another layer of difficulty. HA-based nanomedicines involve new materials and modifications that require extensive testing for safety, stability, and effectiveness. Regulatory approval can be a slow and costly process, especially since there are no standardized guidelines for nanoparticle therapies, further delaying their path to market [119].

## 8. Future Prospectives

HA-based drug delivery systems have great potential, but several challenges still need to be addressed before they can be widely used. Producing HA NPs consistently and at a large scale is one major limitation, requiring further optimization. Additionally, long-term studies are needed to fully understand how safe and compatible these systems are with the human body.

Ongoing research focuses on balancing modifications for stability and functionality without compromising safety or increasing costs. Collaboration between chemists, biologists, and clinicians is essential to overcoming these hurdles for future clinical success. Continued research and development are likely to yield new formulations and applications, further enhancing the therapeutic potential of HA-based technologies. From improving the management of chronic diseases to enabling more precise and effective treatments, HA is set to play a pivotal role in the next generation of medical advancements.

## 9. Conclusions

Hyaluronic acid’s unique properties and versatility make it an invaluable tool in modern medicine. Its applications in drug delivery, particularly for ocular and cancer treatments, showcase its potential to revolutionize therapeutic approaches. As research progresses, HA-based nanoparticles will likely become integral to developing more effective, targeted, and patient-friendly treatments. This review highlights the current innovations and future directions in HA-based drug delivery systems, emphasizing their transformative impact on healthcare.

## Figures and Tables

**Table 2:** Hyaluronic acid-based nanoformulations for drug delivery.

Nanoformulations	Components	Type of Nanodelivery	Type of Study	Reference
HA NPs (doxorubicin and cisplatin)	Hyaluronic acid nanoparticles	CD44 receptor-targeted allograft breast cancer in inoculated mice	In vivo/(4T1 cells into the mouse mammary fat pad)	[66]
HA-conjugated NPs for the delivery of siRNA	Hyaluronic acid-conjugated mesoporous silica nanoparticles	Delivery of siRNA against TWIST protein enhances sensitivity to cisplatin in ovarian cancer	In vivo/epithelial ovarian cancer mice model	[67]
Doxorubicin-loaded dual-functional HA NPs	Hyaluronic acid modified with glycyrrhetinic acid and L-histidine	Liver-targeted and pH-responsive drug delivery	In vitro/HepG2 liver cell line	[68]
HA NPs coloaded with sorafenib and cisplatin	Sodium hyaluronate (MW ¼ 10–20 kDa)	pH-responsive nanoparticles for hepatocellular carcinoma	In vitro/HepG2 cells for HepG2 tumor-bearing mice and in vivo	[69]
HA-modified chitosan nanoparticles loaded with siRNA	Deacetylated chitosan (92%); HA molecular weight is 35 kDa, and sodium tripolyphosphate is also used	Carrier for siRNA delivery and targeted therapy for non-small cell lung cancer expressing CD44	In vivo/A549 cells expressing receptor CD44 and tumor-bearing mice	[70]
HA-modified Inorganic NPs	Hyaluronic acid-modified Au–Ag alloy	Effective tumor radio-sensitization targeting activated hydroxyl radicals (•OH)	In vitro/4T1 breast overexpressing CD44 receptor	[71]
β-Cyclodextrin-modified HA Supramolecular self-assemblies	HA, MW10 KDa, curcumin, succinic anhydride Hydrogen peroxide (3–dimethylaminopropyl)−3–ethylcarbodiimide hydrochloride N-hydroxysuccinimide 4–dimethylaminopyridine	Supramolecular self-assemblies with active targeting	In vitro/prostate cancer (PC—3) cell line	[72]
HA-based NPs	Modified HA with cationic polyethyleneimine (PEI)	Macrophage-targeted MicroRNA/inflammation, tumors, autoimmune disorders, and tissue repairs	In vitro	[46]
pH-sensitive HA-Based NPs	HA (MW = 1.0 × 10^5^ Da) Doxorubicin and cisplatin	CD44-targeted anti-breast cancer drug delivery system	In vitro and in vivo murine CD44 + mammary carcinoma cells (4T1)	[73]
Oxygen-resupply HA NPs	HA and chlorin e6 and cisplatin	Cisplatin cross-linking, chlorin e6 (Ce6) loading, pH sensitivity, active targeting, and oxygen resupply nanoplatform	In vitro and in vivo photodynamic therapy under 660 nm laser	[74]
HA NPs	HA with different molecular weight	Protein delivery NPs/myoglobin, model neutral protein	In vitro/cells overexpressing HA receptors, such as CD44	[75]
ATP-sensitive DNA polyplexes coated with HA	Hyaluronate sodium, 95%, low molecular weight protamine, phospholipids, and doxorubicin	Targeted ligand for tumor marker CD44	In vitro/MDA-MB−231 breast cancer cells	[43]
HA-modified metal–polyphenol	HA-modified ferrous baicalein, Zn(CH_3_COO)_2_, MnCl_2_·4H_2_O, catechol, dopamine, resorcinol epigallocatechin, polyvinylpyrrolidone PVP	Photothermal nanoparticles for cancer therapy	In vitro	[76]
Surface-functionalized HA-coated thiolated chitosan NPs	Chitosan (Cs) (low molecular weight (50–190 kDa, with 75–80% deacetylation), thioglycolic acid (TGA), and tripolyphosphate polyanions (TPP) 1-ethyl-3-3(3-dimethylamino propyl carbodiimide hydrochloride vincristine	Targeted drug delivery for prostate cancer	In vitro/prostate epithelial cells (HPrEC) and	[77]
HA/nanofiber	Hyaluronic acid/polyvinyl alcohol electro-spun and Plantago major extract	Smart wound dressings	In vitro	[78]
HA- PLGA) magnetic NPs	Hyaluronic acid (HA)-modified poly (lactic-co-glycolic acid) (PLGA) and cisplatin	NIR-responsive chemo-photothermal combination	In vivo implanted U87 glioblastoma cells in nude mice	[79]
HA-coated gold NPd	HAs with different molecular weights were produced by e-beam irradiation and employed as coating materials for AuNPs	Hyaluronic acid-coated gold nanoparticles as a controlled drug delivery system for sulfasalazine	In vitro c2c12 cell line, a subclone of the mouse myoblast cell line	[80]
HA-coated, thiolated chitosan-encapsulated NPs	HA-thiolated chitosan and cisplatin	Cervical carcinoma CD44-targeted delivery and sustained release	In vitro drug release kinetics In vitro cervical cancer cells HeLa	[47]
Curcumin-loaded γ-cyclodextrin-grafted HA nanoassimblies	Mono-6-deoxyl-6-ethylenediamino-γ-cyclodextrin was grafted to a high molecular weight HA polymer by carbodiimide	Wound healing and inflammatory activity of hyaluronic acid and curcumin	In vitro/human dermoblast cell response to THP−1	[81]
HA-coated chitosan NPs	Chitosan (medium molecular weight, 190–310 kDa), HA Mw = 1500 kDa (C_7_H_8_O_2_), hydrogen peroxide	Carrier for the enzyme/prodrug complex based on horseradish peroxidase/indole-3-acetic acid for bladder cancer cells	In vitro/bladder cancer cell line (T24)	[82]
HA-coated Niosomes	HA and cationic surfactant	Targeted nanocarrier	In vitro/MCF-7 breast cancer cell line	[83]
Nitric oxide-scavenging HA NPs	Derivative of o-phenylenediamine (o-PD) with good NO-scavenging capability is designed, synthesized and chemically conjugated to HA	NO-scavenging and anti-inflammatory for the treatment of osteoarthritis	In vivo/osteoarthritis animal model	[84]
CD44-targeting HA–selenium NPs	Hyaluronic acid and selenic acid	CD44-targeting scavenging ROS and suppressing inflammatory responses in the injured spinal cord	In vitro/microglia cells (BV2) upon lipopolysaccharide-induced inflammation	[85]
HA-stabilized Ag NPs	Hyaluronic acid and silver	Non-toxic nanocatalysts in the oxidation of morin	In vitro	[86]
HA-functionalized DDAB/PLGA NPs for oral delivery of magnolol	HA, dimethyl-dioctadecyl-ammonium bromide (DDAB), and poly lactic-co-glycolic acid (PLGA) hybrid nanoparticle	The treatment of ulcerative colitis	In vitro	[87]
